# Local Actions of Melatonin in Somatic Cells of the Testis

**DOI:** 10.3390/ijms18061170

**Published:** 2017-05-31

**Authors:** Mónica Beatriz Frungieri, Ricardo Saúl Calandra, Soledad Paola Rossi

**Affiliations:** Instituto de Biología y Medicina Experimental, Consejo Nacional de Investigaciones Científicas y Técnicas (CONICET), Vuelta de Obligado 2490, Buenos Aires 1428, Argentina; ricardoscalandra@gmail.com (R.S.C.); soledadrossi3@hotmail.com (S.P.R.)

**Keywords:** melatonin, testis, androgen production, oxidative stress, inflammation, infertility, Leydig cells, Sertoli cells, mast cells, macrophages

## Abstract

The pineal hormone melatonin regulates testicular function through the hypothalamic-adenohypophyseal axis. In addition, direct actions of melatonin in somatic cells of the testis have been described. Melatonin acts as a local modulator of the endocrine activity in Leydig cells. In Sertoli cells, melatonin influences cellular growth, proliferation, energy metabolism and the oxidation state, and consequently may regulate spermatogenesis. These data pinpoint melatonin as a key player in the regulation of testicular physiology (i.e., steroidogenesis, spermatogenesis) mostly in seasonal breeders. In patients with idiopathic infertility, melatonin exerts anti-proliferative and anti-inflammatory effects on testicular macrophages, and provides protective effects against oxidative stress in testicular mast cells. Consequently, melatonin is also involved in the modulation of inflammatory and oxidant/anti-oxidant states in testicular pathology. Overall, the literature data indicate that melatonin has important effects on testicular function and male reproduction.

## 1. Introduction

Male reproductive functions are regulated by the luteinizing hormone (LH) and the follicle stimulating hormone (FSH) secreted by the pituitary gland. The hypothalamic gonodotropin releasing hormone (GnRH) and the gonadotropin inhibitory hormone (GnIH), via hypothalamic-pituitary portal veins, are carried directly to the anterior pituitary gland where they bind to specific receptors modulating the release of LH and FSH. LH binds to Leydig cells in the testicular interstitium stimulating androgen production. FSH binds to Sertoli cells in the seminiferous tubules stimulating their secretory activity and the subsequent normal progression of spermatogenesis [1,2]. Particularly, in rodents, pituitary control of the testis also involves the hormone prolactin, which binds to Leydig cells and regulates the number of LH receptors [3].

Numerous species experience seasonal changes in their reproductive activities, which are dependent on the photoperiod length [4]. Thus, in seasonal breeders, the function of the hypothalamic-pituitary-testicular axis undergoes cyclic variations consisting of periods of activation (during sexual maturation and at the beginning of each annual breeding season), and inactivation during the hibernation period. The photoperiod influences seasonal breeders through changes in melatonin secretion by the pineal gland. The light signal is received by the photoreceptors of the retina and transferred from the eyes to the pineal gland through a circuitous connection of neurons involving retinohypothalamic fibers, the suprachiasmatic nuclei, hypothalamus-pineal fibers, and the peripheral sympathetic nervous system, leading to the regulation of melatonin synthesis and secretion [5,6,7]. In long-day (LD) seasonal breeders such as the hamster, the duration of daily melatonin secretion is negatively correlated with the number of hours that the seasonal animal is exposed to daylight, and therefore, light signal is interpreted as pro-gonadotropic. On the contrary, light signal results in being anti-gonadotropic in short day (SD) animals such as several breeds of sheeps and goats. SD animals are sexually active during the shortest days of the year when melatonin levels are maximal in terms of their nocturnal duration [8]. Finally, in non-seasonal breeders like humans, which are sexually active all through the year irrespective of the season, the role of melatonin in male reproduction is still poorly understood [9].

Melatonin exerts its effects via specific receptors coupled to G proteins and characterized by a seven transmembrane-spanning domain. Cloning studies identified three subtypes of membrane melatonergic receptors: Melatonergic type 1 (MT1) receptors, which are expressed in the suprachiasmatic nucleus of the hypothalamus and pars tuberalis of the pituitary, melatonergic type 2 (MT2) receptors mainly located in the brain and retina, and MT3 receptors, which are not expressed in mammals but were found in fish, amphibians, birds and chickens [10]. *Mt1* and *Mt2* receptors are expressed in rodent testis including mice, rats and hamsters [11,12,13,14]. Melatonin also exerts its effects by binding to orphan nuclear receptors (the retinoid-related orphan nuclear hormone receptor RZR/RORα and the X-linked melatonin-related orphan receptor GPR50) and intracellular proteins such as calmoduline [10].

In males, besides its effect on the synthesis and secretion of the hypothalamic GnRH and the adenohypophyseal gonadotropin hormones, melatonin released from the pineal gland to the circulation is taken up by the testis, where it directly modulates testicular activity [8,15,16,17]. In addition, testes are able to synthesize melatonin [18,19,20].

It has been shown that, by binding specific receptors, melatonin directly regulates testosterone secretion [12,21,22,23], increases the responsiveness of Sertoli cells to FSH during testicular development [24] and modulates cellular growth, proliferation, and the secretory activity of several testicular cell types [25]. This indolamine also protects the testis against local inflammatory processes and the generation of reactive oxygen species (ROS). In this sense, melatonin treatment reduces the severity of the testicular damage generated in animal models with hyperlipidemia, induced gonadal torsion, artificial varicocele or toxicity provoked by exogenous chemicals such as anti-cancer drugs or environmental toxicants [26,27,28,29,30,31,32]. Serum levels of melatonin decrease with advancing age. In this context, Rocha et al. [33] have proposed that melatonin supplementation in elderly men may be an effective therapy for diabetic patients facing impairment of fertility. Melatonin also seems to protect human spermatozoa from apoptosis [34]. In fact, low melatonin levels have been associated with reduced sperm motility and abnormal sperm progression [35,36]. Moreover, melatonin serum and seminal plasma levels in infertile patients suffering from oligoasthenozoospermia or non-obstructive azoospermia are significantly reduced compared with those quantified in fertile men [36].

The interest in a possible role of melatonin in testicular function led us to readdress this issue and we therefore studied the impact of this indolamine in somatic cells of the testis.

This review summarizes the outcome of these studies as well as the current state of knowledge with regards to the local effects exerted by melatonin on Leydig cells, Sertoli cells and testicular immune cells. In particular, data highlight the relevance of melatonin in testes of men with idiopathic infertility.

## 2. Melatonin in Leydig Cells

The expression of the two key enzymes involved in melatonin synthesis, arylalkylamine*-N-*acetyltransferase (AANAT) and *N-*acetylserotonin*-O-*methyltransferase (ASMT) has been reported in ram Leydig cells [20]. These enzymes catalyze the conversion of serotonin (5-HT) into melatonin. In this context, we have previously described that, at least in the hamster, Leydig cells contain and synthesize 5-HT [37,38].

Initial characterization of melatonin receptors in Leydig cells by 2-[125I] iodomelatonin-binding studies and by pharmacological assays using luzindole (a MT1/MT2 receptor antagonist) was unable to discriminate among different subtypes of melatonin receptors [21,39]. Further experiments using reverse transcription polymerase chain reaction (RT-PCR) and Western blot techniques characterized the presence of *Mt1*/MT1 but not *Mt2*/MT2 expression in hamster Leydig cells [12].

To evaluate a potential physiological role of melatonin in Leydig cells, early studies examined the in vitro effect of this indolamine on steroidogenesis. For instance, Wu et al. [21] found that in MA-10 mouse Leydig tumor cells, this indolamine directly inhibited the human chorionic gonadotropin- (hCG-) or dibutyryl-cyclic adenosine monophosphate (db_c_-AMP)-stimulated production of progesterone (the major steroid synthesized by this cell line) [21]. In rat Leydig cells, Valenti et al. [40] described a dose-dependent inhibition of testosterone release in the presence of melatonin. However, no significant changes in the level of serum testosterone were found in melatonin-treated rats [22]. Furthermore, data from our group revealed that physiological concentrations of melatonin exert a direct inhibitory effect on hCG-stimulated cAMP and testosterone production in Leydig cells from reproductively active Syrian (golden) hamsters (*Mesocricetus auratus*) [12].

Reproductive activity in Syrian hamsters is restricted to spring and summer. Under artificial light conditions, male adult hamsters need to be kept under an LD photoperiod (14 h light, 10 h dark) to remain sexually active. Exposure to a SD photoperiod (less than 12.5 h of light per day) for a period of 8–16 weeks results in a marked testicular regression as a consequence of a severe fall in LH, FSH and prolactin circulating levels and a decline in blood and gonadal concentrations of testosterone, its hormonal precursors, and its metabolites [1,41]. Besides the absence of stimulating pituitary factors, a negative regulation of steroidogenesis by signals originated within and/or outside the testis contributes to the profound decrease detected in serum androgen concentrations during the involution phase in Syrian hamsters. In this context, melatonin reduced hCG-stimulated 5α-androstane-3α,17β-diol (3α-Diol) secretion in Leydig cells purified from hamsters exposed to a SD photoperiod for 16 weeks. It is important to bear in mind that although testicular and circulating levels of androgens are markedly reduced during the regression period, inactive adult hamster testes released more 5α-reduced compounds (dihydrotestosterone (DHT) and 3α-Diol) than active adult hamster testes, 3α-Diol being the main androgen produced under in vitro conditions [42]. Morphological and histological changes occur in the tubules and the interstitial compartment during testicular regression. Although the number of Leydig cells per testis remains almost constant, a significant decrease in the absolute volume and surface area of Leydig cell organelles such as smooth endoplasmic reticulum and mitochondria has been described [1,43,44]. However, testicular androgen biosynthetic capacity is not reduced. In fact, regressed testes reached an intermediate physiological state between peripubertal and active adult testes (see further details in Frungieri et al. [42]).

In Leydig cells, LH stimulates testosterone production through the cAMP signaling pathway. In addition, endocrine and paracrine factors via cAMP-independent mechanisms have been found to be associated with testosterone secretion regulation [45]. For example, GnRH stimulates androgen secretion via activation of protein kinase C (PKC) and an increment in the cytosolic Ca^2+^ concentrations. Subsequent studies showed that melatonin reduced GnRH-induced testosterone secretion by suppressing the GnRH-dependent release of Ca^2+^ from intracellular stores in rat Leydig cells [46].

Later studies indicated that melatonin regulates the expression of the steroidogenic acute regulatory (StAR) protein and important steroidogenic enzymes. Wu et al. [21] found that melatonin inhibited StAR protein expression in MA-10 mouse Leydig tumor cells. In hamster Leydig cells, melatonin reduced the expression of *StAR*, cytochrome P450 family 11 subfamily A member 1 (*Cyp11a1*), 3β-hydroxysteroid dehydrogenase (*Hsd3b*), and 17β-hydroxysteroid dehydrogenase type III (*Hsd17b3*) [12]. Maitra and Ray [22] also described a significant decrease in the testicular activity of HSD3B and Hsd17b3 in adult rats injected with melatonin. In particular, in Leydig cells of inactive hamsters, melatonin induces the expression of steroid 5α-reductase (*Srd5a*), an enzymatic isoform that plays a crucial role for the testicular conversion of testosterone into the active and nonaromatizable testosterone metabolite DHT, but inhibits the expression of 3α-hydroxysteroid dehydrogenase, an enzyme that catalyzes the interconversion between DHT and 3α-Diol [12]. Consequently, in regressed Leydig cells, melatonin modulates the production of DHT and 3α-Diol by inducing the conversion of non-5α-reduced into 5α-reduced androgens and inhibiting the production of the main androgen 3α-Diol.

In brief, melatonin acts directly on rodent Leydig cells by modulating androgen production via cAMP-dependent and cAMP-independent pathways and regulation of steroidogenic gene expression.

Cross-interactions between the melatonergic system and other systems located in the testis have been described. In this regard, melatonin modulates testosterone production via its interaction with the testicular corticotropin-releasing hormone (CRH) system. CRH is secreted by the hypothalamus and regulates the pituitary–adrenocortical axis [47]. However, the production and secretion of CRH has also been reported as well as the expression of its receptor (*Crh-r1*) in mouse, rat and hamster Leydig cells [12,48,49,50,51]. CRH modulates testosterone production, although controversial findings were published in mouse and rat Leydig cells [48,49]. CRH has been proposed as a negative modulator of hCG-stimulated testicular steroidogenesis in rats. However, the basal production of testosterone and cAMP remained unchanged when rat Leydig cells were incubated in the presence of this hormone [48,49,52]. In mouse Leydig cells, it has been described that CRH increases the basal testosterone production and cAMP concentration without affecting the maximum hCG-stimulated testosterone synthesis [51,53]. Data from our group revealed an inhibitory effect of CRH on gonadotropin-induced cAMP and androgen production in hamster Leydig cells from both reproductively active and inactive animals [12]. Additional experiments demonstrated that the competitive CRH receptor antagonist α-helical CRH (9–41) blocks the inhibitory effect of melatonin on hCG-stimulated cAMP and androgen production in hamster Leydig cells, suggesting that melatonin’s effect on steroidogenesis could take place through the local CRH system [12].

**Figure 1:**
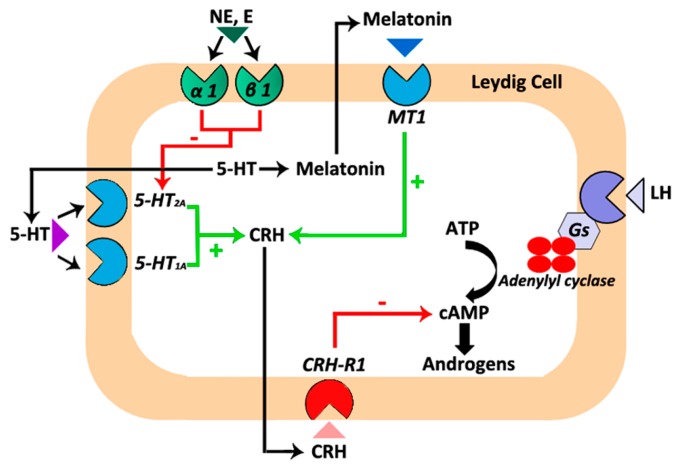
A summary of the interactions between the local melatonergic, serotoninergic, catecholaminergic and corticotropin-releasing hormone (CRH) systems that are involved in the regulation of androgen production in hamster Leydig cells. Melatonin acting through specific melatonergic type 1 (MT1) receptors located in hamster Leydig cells stimulates CRH production [12]. Serotonin (5-HT), the precursor of melatonin, also stimulates CRH production via 5-HT1A and 5-HT2A receptors [38]. Subsequently, CRH through CRH-R1 receptors inhibits human chorionic gonadotropin (hCG)-stimulated androgen production [12,38]. In addition, epinephrine/norepinephrine through α1/β1-adrenergic receptors set up a brake on the inhibitory effect exerted by the 5-HT/5-HT2A receptors/CRH system on androgen production [38]. Green arrows/+ symbolize stimulatory effects. Red arrows/- symbolize inhibitory effects.

Like melatonin, 5-HT inhibits testosterone production in rat and hamster Leydig cells through the CRH system [38,49]. In addition, in hamster Leydig cells, the 5-HT/CRH system remains under the influence of epinephrine and norepinephrine acting through α1/β1-adrenergic receptors. Epinephrine and norepinephrine avoid the stimulatory action exerted by 5-HT/5-HT2A receptors on CRH production. Therefore, epinephrine and norepinephrine decrease intracellular CRH levels and, consequently, increase testosterone production [38]. Overall, interactions between the testicular melatonergic, serotoninergic, catecholaminergic, and CRH systems take part in the modulation of cAMP and testosterone production at least in Leydig cells of the Syrian hamster (Figure 1).

**Figure 2:**
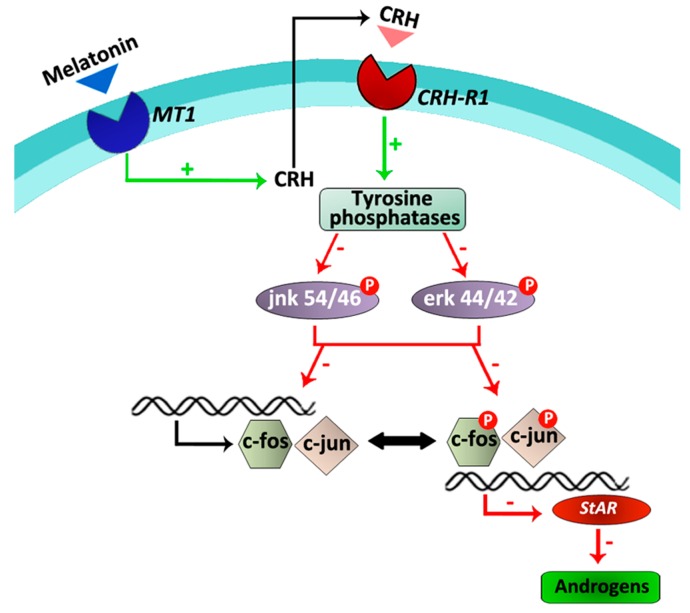
Schematic overview of the melatonin/CRH signaling pathway in hamster Leydig cells. Melatonin locally stimulates CRH production in hamster Leydig cells. Thus, activation of tyrosine phosphatases via *Crh-r1* receptors leads to reduced phosphorylation levels of erk 44/42 and jnk 54/46, down-regulation of c-jun and c-fos expression, inhibition of the transcription factors phosphorylation, decreased expression of *StAR* and consequently, diminished androgen production [12,54]. Green arrows/+ symbolize stimulatory effects. Red arrows/- symbolize inhibitory effects.

Subsequent studies were developed to further establish the initial events of the melatonin/CRH signaling pathway. Results indicated that in hamster Leydig cells, melatonin stimulates the activity of tyrosine phosphatases and reduces the phosphorylation levels of the mitogen activated protein (MAP) kinases, erk and jnk, which directly or indirectly play roles in the regulation of cell cycle, apoptosis, inflammation, cell differentiation and proliferation [54]. Similar actions of melatonin have previously been described in human umbilical vein endothelial cells, hepatoma cells, VSC4.1 motoneurons and spinal cord injury [55,56,57,58]. Phosphorylation and regulation of c-fos transcription is triggered by erk, whereas activation of the jnk pathway depends on c-jun. In this context, melatonin also downregulated c-fos and c-jun expression as well as *StAR* expression in hamster Leydig cells [54]. When tyrosine phosphatases activity, MAP kinases phosphorylation, early immediate genes and *StAR* expression, and testosterone production were evaluated in the presence and absence of the competitive CRH receptor antagonist α-helical CRH (9–41), results revealed that melatonin does not exert a direct role. On the contrary, this indolamine acts indirectly on tyrosine phosphatases, MAP kinases, transcription factors and the steroidogenic pathway via its stimulatory role on the local CRH production [54] (Figure 2).

Taken together, the present results indicate that, at least in hamster Leydig cells, melatonin inhibits androgen production via its interaction with the local CRH system and a regulatory pathway composed of tyrosine phosphatases, MAP kinases and transcription factors.

All components involved in melatonin/CRH-induced downregulation of androgen production in hamster Leydig cells were also identified in testes of infertile men. For instance, testicular biopsies of patients suffering idiopathic infertility contain measurable levels of melatonin and human Leydig cells express *CRH* as well as *MT1* and *CRH-R1* receptors [54]. Because melatonin and CRH target human Leydig cells, it is possible to formulate a conjecture about the participation of these hormones in the modulation of human Leydig cell function in some fertility disorders. Nevertheless, further investigations are required to determine the precise role of melatonin and CRH in the human testes and if the experimental results obtained in hamster Leydig cells can be extrapolated to states of male infertility.

In summary, the literature data indicate that melatonin acts on rodent Leydig cells regulating androgen production.

## 3. Melatonin in Sertoli Cells

The expression of *Mt1* and *Mt2* receptors has been reported in rat and bovine Sertoli cells [33,59].

Sertoli cells are, within the seminiferous tubules, the major transducers of testosterone and FSH signals that are required to support germ cell survival and development [60]. Because Sertoli cells play a key role in spermatogenesis efficiency and fertility [61], several authors lead the efforts to evaluate a possible role of melatonin in the regulation of Sertoli cell function.

Initial studies suggested that melatonin has adverse effects on mice and rats seminiferous tubules [62,63]. Sertoli cells provide energy substrates such as lactate required to fuel germ cell metabolism. It has been described that melatonin decreases basal lactate production, but upregulates the insulin-stimulated lactate generation in rat Sertoli cells [33]. In this context, several biochemical mechanisms may contribute to alterations in lactate production and secretion; one of them is cellular glucose uptake, the main carbon source for lactate synthesis. In Sertoli cells, facilitated diffusion of glucose across plasma membrane is mediated by the glucose transporters GLUT1, GLUT3 and GLUT8 [64,65,66]. It has been shown that melatonin increases GLUT1 protein levels and glucose consumption in rat Sertoli cells [33].

Lactate production also depends on lactate dehydrogenase expression (LDH) and activity. Melatonin decreases LDH protein levels and activity in rat Sertoli cells [33]. Not only lactate but also acetate can be used by Sertoli cells as a source and store of energy. Esterification of acetate to acetyl-CoA depends on acetyl-CoA synthase, while re-conversion of acetyl-CoA to acetate is catalyzed by acetyl-CoA hydrolase [67]. In addition, acetate may follow other paths. Arachidonic acid is formed from an exogenous C18 precursor (presumably derived from linoleic acid) by the addition of a C2 fragment derived from acetate. In rat Sertoli cells, acetate production is upregulated by melatonin [33]. It has been recently proposed that acetate could be essential for the maintenance of an adequate rate of lipid synthesis in developing germ cells [33].

Despite the low oxygen tension that characterizes the testicular micro-environment, the testis remains vulnerable to oxidative stress due to the abundance of highly unsaturated fatty acids and the presence of systems that generate ROS including the mitochondria and a variety of enzymes such as the xanthine- and reduced nicotinamide adenine dinucleotide phosphate (NADPH)-oxidases and the cytochrome P450s [68]. Oxidative stress might lead to an impaired spermatogenesis, and, therefore, to infertility [69]. There are many factors capable of inducing oxidative stress in the testes. For instance, we have recently described that PGD2 increases ROS generation in the mouse TM4 Sertoli cell line [70]. Furthermore, Rocha et al. [33] found that insulin increases the intracellular levels of lactate and alanine in rat Sertoli cells, and, therefore, decreases the lactate/alanine ratio. This ratio reflects the intracellular redox state since the interconversion of pyruvate to lactate and/or alanine is coupled to the re-oxidation of NADH to NAD+. Interestingly, melatonin restores intracellular lactate and alanine levels as well as the lactate/alanine ratio to its baseline values. Thus, at least in insulin-treated rat Sertoli cells, melatonin exerts a modulatory role on the oxidant/anti-oxidant balance maintaining, consequently, the intracellular redox state at control levels.

On the other hand, melatonin has been shown to exert both anti- or pro-inflammatory actions depending on the physio/pathological status. As a rule, anti-inflammatory effects are the most evident under situations of high-grade inflammation. In contrast, pro-inflammatory actions are frequently observed under basal conditions [71,72]. With regard to the above mentioned, unpublished results from our group suggest that melatonin induces the expression of cyclooxygenase 2 (*Cox2*) and lipocalin-type prostaglandin D synthase (*L-pgds*), key enzymes in the synthesis of prostaglandins, in murine TM4 Sertoli cells [73]. Prostaglandins play a crucial role in the generation of the inflammatory response. Consequently, melatonin targeting Sertoli cells might contribute to the generation of inflammatory responses in the testis.

Melatonin also affects Sertoli cell growth and proliferation. In bovine Sertoli cells, melatonin decreases the mRNA levels of *P21*, a potential inhibitor of G1 cyclin-dependent kinases [59]. Moreover, melatonin promotes spermatogonial stem cells (SSCs) proliferation by stimulating glial cell line-derived neurotrophic factor (GDNF) production in Sertoli cells [74]. Melatonin also upregulates the expression of spermatogenesis-related genes, including *Cyclin D1*, *Cyclin E*, *Pdgfa*, *Dhh*, *Occludin*, and *Claudin* in bovine Sertoli cells, and, therefore, it might affect spermatogenesis and the blood–testis barrier [59].

It has been reported that in bovine Sertoli cells, melatonin significantly increases inhibin βA, inhibin βB and inhibin α mRNA expression as well as the secreted levels of inhibin B, a marker of Sertoli cell damage and spermatogenic disturbance [59]. In addition, melatonin increases the responsiveness of the Sertoli cell to FSH during gonadal development, which may help to prevent testicular damage [24].

From the aforementioned data, it is clear that melatonin exerts a plethora of functions in the Sertoli cell regulating its growth, proliferation, oxidant/anti-oxidant status, the energy metabolism and the production of prostaglandins. Because male fertility and the process of spermatogenesis are strongly dependent on Sertoli cells function, we can speculate that melatonin acts as an important modulator in the progression of germ cells to spermatozoa.

## 4. Melatonin in Testicular Immune Cells

A large number of reports implicate melatonin as an immunomodulatory compound. Recently, the role of melatonin on the immune system has been described as that of a buffering agent, acting as a stimulant under basal or immunosuppressive conditions or as an inhibitory factor in the presence of exacerbated immune responses, such as acute inflammation [71].

Melatonin receptors are detectable in T helper cells, lymphocytes, granulocytes, mast cells and monocytes/macrophages [75,76,77,78].

The testis is considered an immune-privileged organ. However, immune cells gain access to the testis, and some of them also undergo local proliferation. Leukocytes, including T cells, natural killer (NK) cells, mast cells, eosinophils and macrophages were localized in the testis [79]. Among immune cells, mast cells and macrophages are best known for their role in inflammatory processes. Nevertheless, in the testis, mast cells and macrophages are also involved in the regulation of steroidogenesis, Sertoli cell activity, germ cell survival, and the generation of fibrosis in the wall of the seminiferous tubules [25,80,81,82,83,84,85,86,87].

The human testis contains melatonin as well as macrophages and mast cells expressing melatonergic receptors [25]. This issue led us to investigate a potential role of melatonin in the regulation of the testicular macrophage and mast cell populations. Data from our group described that the number of macrophages is significantly higher in testes of patients with hypospermatogenesis or Sertoli cell only (SCO) syndrome than in gonads of healthy men [84]. Moreover, testicular melatonin concentrations inversely correlate with the number of macrophages in biopsies from infertile patients. Subsequent studies established that melatonin decreases cell density and the expression of the proliferating cell nuclear antigen (PCNA) without affecting cell viability in both non-human testicular macrophages and human non-testicular macrophages [25].

Melatonin also inhibited the expression of the pro-inflammatory cytokines *TNFα* and *IL1β*, as well as the expression of *COX2* in human non-testicular and testicular non-human macrophages [25]. It has been already proposed that melatonin regulates the immune system by affecting cytokine production in immunocompetent cells. In this context, melatonin increases the production of IL-2, IFNγ and IL-6 in human mononuclear cells [88], the secretion of IL-1, IL-6, IL-12 and TNFα in monocytes [89], the production of IFNγ by Th1 cells [88] as well as the expression of IL-2 and IL-12 in NK cells [88,90,91].

Additionally, Pawlak et al. [92] described that a physiological concentration of melatonin significantly increases the phagocytic index in testicular macrophages of rats via a Ca^2+^-dependent mechanism.

Hence, melatonin targeting testicular macrophages plays local pro-phagocytic, anti-proliferative and anti-inflammatory roles.

Regarding mast cells, Izzo et al. [93] described that melatonin decreases testicular mast cell population in the frog *Pelophylax esculentus*. However, although mast cell population is significantly higher in testes of infertile patients than in healthy men [82], no correlation was seen between testicular melatonin concentrations and the number of mast cells in testes of patients suffering from hypospermatogenesis or SCO syndrome [25].

Mast cells secrete a plethora of potent mediators including proteases. Mast cells proteases are the most precise markers of mast cells population heterogeneity. Two types of mast cells have been recognized. Mast cells containing tryptase together with chymase, cathepsin-G like protease, and carboxypeptidase (MCTC), and mast cells which contain tryptase (MCT) but lack the other neutral proteases present in MCTC cells [94]. Therefore, the serine protease tryptase is of special interest because it is expressed in almost all populations of mast cells. The expression of tryptase and chymase was described in testicular mast cells of infertile patients [25]. Furthermore, a direct correlation between testicular melatonin concentrations and the expression of these serine proteases in human testicular biopsies of patients with hypospermatogenesis and SCO syndrome was reported [25].

The mast cells product tryptase decreased motility in human spermatozoa while chymase showed no such effect [95]. In peritubular cells of the human testis, tryptase induced *COX2* expression, 15d-PGJ2 production and, subsequently, fibrosis of the tubular wall [96,97,98]. Tryptase also altered the microenvironment in the human testes with regards to neurotrophin actions and the production of the extracellular matrix protein decorin [99,100].

Hence, melatonin seems to stimulate the expression of the mast cell proteases tryptase and chymase involved in the modulation of the extracellular matrix protein decorin production, sperm motility and the generation of fibrotic events, and, consequently, this indolamine acting on the local mast cell population might participate in the regulation of testicular functionality and male fertility.

**Figure 3:**
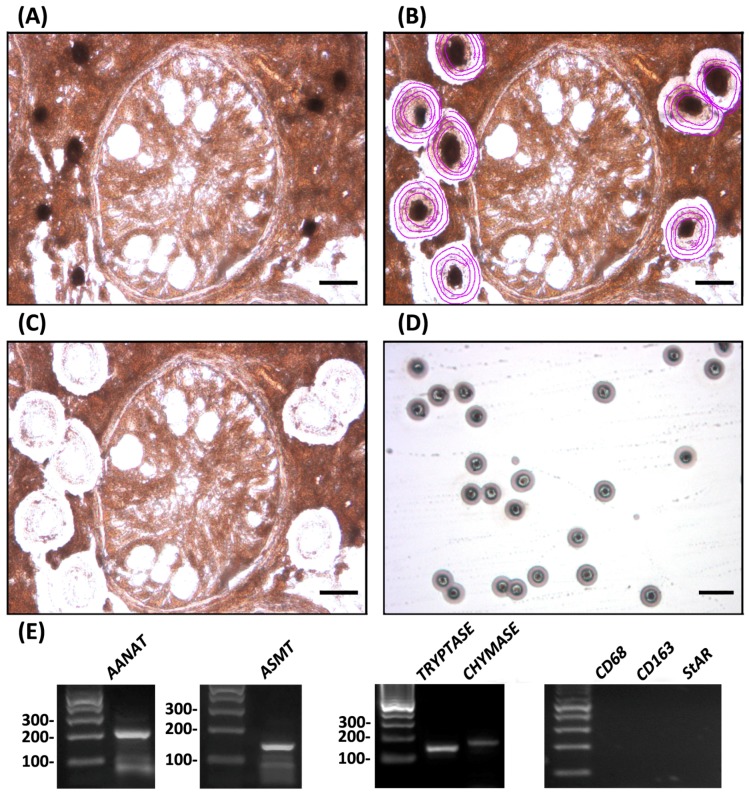
Using laser capture microdissection, tryptase-immunoreactive mast cells were isolated from testicular biopsies of patients suffering from hypospermatogenesis or Sertoli cell only (SCO) syndrome, and subjected to RT-PCR studies. Panels depict the same specimen before laser microdissection (**A**), after ultraviolet laser delimitation (purple circles) of tryptase-immunoreactive mast cells (**B**) and after infrared laser microdissection of target cells (**C**). Bar, 25 μm; (**D**) depicts tryptase-immunoreactive mast cells captured into the cap of a microfuge tube. Bar, 100 μm. A monoclonal mouse anti-human tryptase antibody (1:50, DAKO) was used; (**E**) Expression of the melatonin-synthesizing enzymes *AANAT* (234 bp) and *ASMT* (149 bp) was detected in the microdissected tryptase-immunoreactive mast cells by RT-PCR assays performed using the following oligonucleotide primers: *AANAT*, first set 5′-GGGACAAGGGAGACTTA and 5′-TCAGCAGCCGCTGTTCC, heminested set 5′-CCGGCAGCAGGGCAGGGG and 5′-TCAGCAGCCGCTGTTCC; *ASMT*, 5′-GAGACGAGGGGAGGAAAAGC and 5′-GTCGTCCTTCTGCTACCT. Expression of the serine protease tryptase (142 bp) and chymase (168 bp) was used as positive controls. Expression of *CD68* and *CD163* (macrophage markers) and *StAR* (Leydig cell marker) in tryptase-immunoreactive mast cells was not found, indicating that the material employed in laser capture microdissection was not contaminated with other testicular cell populations. Expression of positive and negative controls was also determined by RT-PCR assays using oligonucleotide primers from Rossi et al. [25]. PCR products were separated on 2% agarose gels and visualized with ethidium bromide. The identity of the cDNA products was confirmed by sequencing, using a fluorescence-based dideoxy sequencing reaction and an automated sequence analysis on an ABI 373A DNA sequencer. The gene expression profile shown is representative of independent analyses performed in six different mast cell preparations (hypospermatogenesis, *n* = 3; SCO, *n* = 3) that showed comparable results. The technique was performed following the procedure described in the materials and methods section from Rossi et al. [25].

To evaluate the relevance of testicular mast cells as a potential source of melatonin, we isolated tryptase- and chymase-immunoreactive mast cells from testes of patients suffering from idiopathic infertility. Subsequent studies demonstrated that testicular mast cells express the melatonin-synthesizing enzymes *AANAT* and *ASMT* (Figure 3).

Under physiological conditions, the immunosuppressive testicular microenvironment protects germinal cells from being attacked by the immune system. However, in inflammatory conditions with increased density of resident macrophages and mast cells [82,84], this tolerance is disrupted and immune cells and their mediators respond to germinal cell self-antigens, inducing damage to the germinal epithelium [101]. The testicular damage induced by oxidative stress is currently considered one of the most important causes of impaired testicular function. Human testicular mast cells express the anti-oxidant enzymes *SOD1*, *PXR1* and *CAT* [25]. A positive correlation between testicular melatonin concentrations and the expression of these anti-oxidant enzymes was found in biopsies of infertile men [25].

On the other hand, oxidative stress might play a pivotal role in apoptosis. In fact, it has been suggested that oxidative stress and apoptosis may be functionally linked. In the testis, an increasing amount of evidence suggests that oxidative stress can induce apoptosis in germ cells [102]. In agreement with this assumption, melatonin testicular concentrations showed a negative correlation with the pro-apoptotic *BAX/BCL-2* ratio in biopsies of infertile patients [25]. Consequently, the anti-oxidant role of melatonin on testicular mast cells seems to be associated with anti-apoptotic events.

These results highlight a potential anti-oxidant and anti-apoptotic effect of melatonin on testicular mast cells that is in line with numerous reports pointing out the ability of this indolamine to act as a protector against free radical damage [103,104,105].

In summary, testicular macrophages and mast cells are targets of melatonin. This indolamine exhibits anti-proliferative and anti-inflammatory actions on testicular macrophages, while melatonin might provide protective effects against oxidative stress and apoptosis in tryptase- and chymase-positive human testicular mast cells. Therefore, it is plausible to hypothesize about a potential biological relevance of melatonin acting as an immunomodulatory compound in the pathogenesis or maintenance of some states of infertility in humans.

## 5. Conclusions

Regarding testicular non-germ cells, a melatonergic system has been described in the two key somatic cell types of the testis: Leydig and Sertoli cells. Melatonin mainly acts as a local modulator of the endocrine activity in Leydig cells, while it regulates growth, proliferation, energy metabolism, oxidative stress, and the occurrence of inflammatory processes in Sertoli cells. These results, pointing out a crucial role of melatonin in the modulation of the physiological activity of testicular endocrine cells, are summarized in Table 1.

Furthermore, the existence of melatonergic receptors in testicular immune cells (mast cells and macrophages) showing a significant increase in their population number in some idiopathic pathologies, strongly suggests the importance of melatonin setting up a brake in the development of local inflammatory, oxidant and apoptotic events that might further compromise testicular function in patients with idiopathic infertility.

Currently, melatonin is commonly prescribed to treat sleep disorders. It is considered a safe drug. However, in spite of the lack of apparent side effects, the use of melatonin in the treatment of other pathologies is still under discussion because several health benefits have only been attributed to pharmacological doses of this neurohormone.

At present, the majority of infertile men show disorders either untreatable or treatable with drugs of questionable effectiveness. Bearing in mind the research summarized in this review suggesting that melatonin therapy may improve male reproductive potential, the impact of this indolamine on male (in)fertility and/or its future as a potential therapeutic target should be further considered.

Collectively, literature reports crucial roles of melatonin on testicular function. Therefore, future advances in the knowledge of the role played by melatonin and its receptors in the human testis will clarify the beneficial and/or disadvantageous effects of this indolamine for the clinical practice.

## Figures and Tables

**Table 1:** Effects of melatonin on the key somatic cells of the testis: Leydig and Sertoli cells.

Cell Types	Species/Cell Lines	Melotonin Effects		Receptor Types	Melatonin Concentrations	References
Leydig cell	mouse	Inhibition of steroid production	Inhibition of *StAR* protein expression	*Mt1 and/or Mt2*	10 nM to 1 µM	[21]
	MA-10		Inhibition of progesterone production	*Mt1 and/or Mt2*	10 nM to 1 µM	[21]
	Rat	Inhibition of steroid production	Inhibition of androgen production	-	4 pM to 4 µM	[40]
			Inhibition of GnRH-dependent intracellular Ca^2+^release	-	0.2 pM	[46]
	Hamster	Inhibition of steroid production	Stimulation of CRH production	*Mt1*	1 and 10 µM	[12,54]
			Stimulation of tyrosine phosphatases activity	*Mt1*	1 and 10 µM	[54]
			Inhibition of erk and jnk phosphorylation	*Mt1*	1 and 10 µM	[54]
			Downregulation of c-fos and c-jun expression	*Mt1*	1 and 10 µM	[54]
			Inhibition of *StAR*, *Hsd3b* and *Hsd17b*3 expression	*Mt1*	1 and 10 µM	[12,54]
			Inhibition of cAMP generation	*Mt1*	1 µM	[12]
			Inhibition of androgen production	*Mt1*	100 pM to 1 µM	[12,54]
Sertoli cell	Rat	Regulation of energy metabolism	Inhibition of basal lactate production	*Mt1 and Mt2*	1 mM	[33]
			Upregulation of the insulin-stimulated lactate generation	*Mt1 and Mt2*	1 mM	[33]
			Stimulation of GLUT1 protein expression and glucose consumption		1 mM	[33]
			Inhibition of LDH protein expression and activity	*Mt1 and Mt2*	1 mM	[33]
			Stimulation of acetate production		1 mM	[33]
		Prevention of testicular damage	Regulation of intracellular redox state	*Mt1 and Mt2*	1 mM	[33]
	Hamster	Prevention of testicular damage	Stimulation of the responsiveness to FSH during testicular development	-	25 µg daily injection (1 to 15 wk)	[24]
	Bovine	Stimulation of cell growth/proliferation	Downregulation of mRNA *P21* expression	*Mt1 and Mt2*	0.3 to 1 nM	[59]
		Regulation of spermatogenesis	Upregulation of *Cyclin D1*, *Cyclin E*, *Pdgfa*, *Dhh*, *Ocludin* and *Claudin* expression	*Mt1 and Mt2*	0.3 to 1 nM	[59]
	Goat	Stimulation of SSCs proliferation	Stimulation of GDNF production	*Mt1 and Mt2*	1 nM and 1 µM	[73]

Mt1: melatonergic type 1 receptor. Mt2: melatonergic type 2 receptor. StAR: steroidogenic acute regulatory. GnRH: gonodotropin releasing hormone. CRH: corticotropin-releasing hormone. Hsd3b: 3β-hydroxysteroid dehydrogenase. Hsd17b3: 17β-hydroxysteroid dehydrogenase type III. cAMP: cyclic adenosine monophosphate. LDH: lactate dehydrogenase. FSH: follicle stimulating hormone. SSCs: spermatogonial stem cells. GDNF: glial cell line-derived neurotrophic factor. wk: weeks.
